# A snapshot review on materials enabled multimodal bioelectronics for neurological and cardiac research

**DOI:** 10.1557/s43580-023-00645-8

**Published:** 2023-09-22

**Authors:** Mabel Bartlett, Mengdi He, Daniel Ranke, Yingqiao Wang, Tzahi Cohen-Karni

**Affiliations:** 1 Department of Biomedical Engineering, Carnegie Mellon University, Pittsburgh, PA 15213, USA; 2 Department of Materials Science and Engineering, Carnegie Mellon University, Pittsburgh, PA 15213, USA

## Abstract

Seamless integration of the body and electronics toward the understanding, quantification, and control of disease states remains one of the grand scientific challenges of this era. As such, research efforts have been dedicated to developing bioelectronic devices for chemical, mechanical, and electrical sensing, and cellular and tissue functionality modulation. The technologies developed to achieve these capabilities cross a wide range of materials and scale (and dimensionality), e.g., from micrometer to centimeters (from 2-dimensional (2D) to 3-dimensional (3D) assemblies). The integration into multimodal systems which allow greater insight and control into intrinsically multifaceted biological systems requires careful design and selection. This *snapshot review* will highlight the state-of-the-art in cellular recording and modulation as well as the material considerations for the design and manufacturing of devices integrating their capabilities.

## Introduction

Understanding the complex physiology and functions of vital organs, such as the brain and heart, is crucial for developing therapeutic strategies for neurological disorders and cardiovascular diseases [1–3]. The major functional units of the neural and cardiac systems, neurons, and cardiomyocytes, respectively, are excitable cells that communicate via ionic fluxes across the cell lipid bilayer membrane, leading to rapid electric potential changes across a membrane called action potentials [4]. Many neurological and cardiac dysfunctions are linked to impaired electrical activity at the cellular and/or network level, requiring the analysis and understanding of their electrical signals [5, 6].

Bioelectronic devices aim to investigate and modulate these electrical signals to understand various disease states and develop therapeutic interventions [6, 7]. The field of bioelectronics, utilizing electronic devices in living systems, applies advances in tissue engineering, engineered materials, and microfabrication technologies to establish controlled microenvironments and implement input/output components for effective monitoring and modulation of cellular behaviors [1–3, 8]. To better achieve this in a closed-loop manner, it is necessary to develop bioelectronics with multiple functionalities that allow simultaneous recording and stimulation of neural or cardiac activities. The key components of such multimodal bioelectronic platforms include electrophysiology recording, chemical sensing, mechanical recording, and electrical and/or optical stimulation [1–3, 9]. Over the past decade, various bioelectronic platforms with different combinations of modalities have been developed to understand neural functions [1] and facilitate cardiac therapeutics [2], expanding the capabilities of bioelectronic devices to contain two or more functionalities on one platform. Concurrently, novel material systems are being developed to be used in micro/nanobioelectronics to improve the device properties and performance.

Here, we review the recent advances in multimodal bioelectronics and highlight key design and fabrication considerations essential to application-driven engineering of future devices. Firstly, we introduce the four main input/output modalities (electrophysiological recording, mechanical recording, electrical stimulation, and optical stimulation) and their working mechanisms and highlight the materials and structure designs to improve their performance, e.g., the sensitivity of stimulation or longevity of recording.

**Fig. 1:**
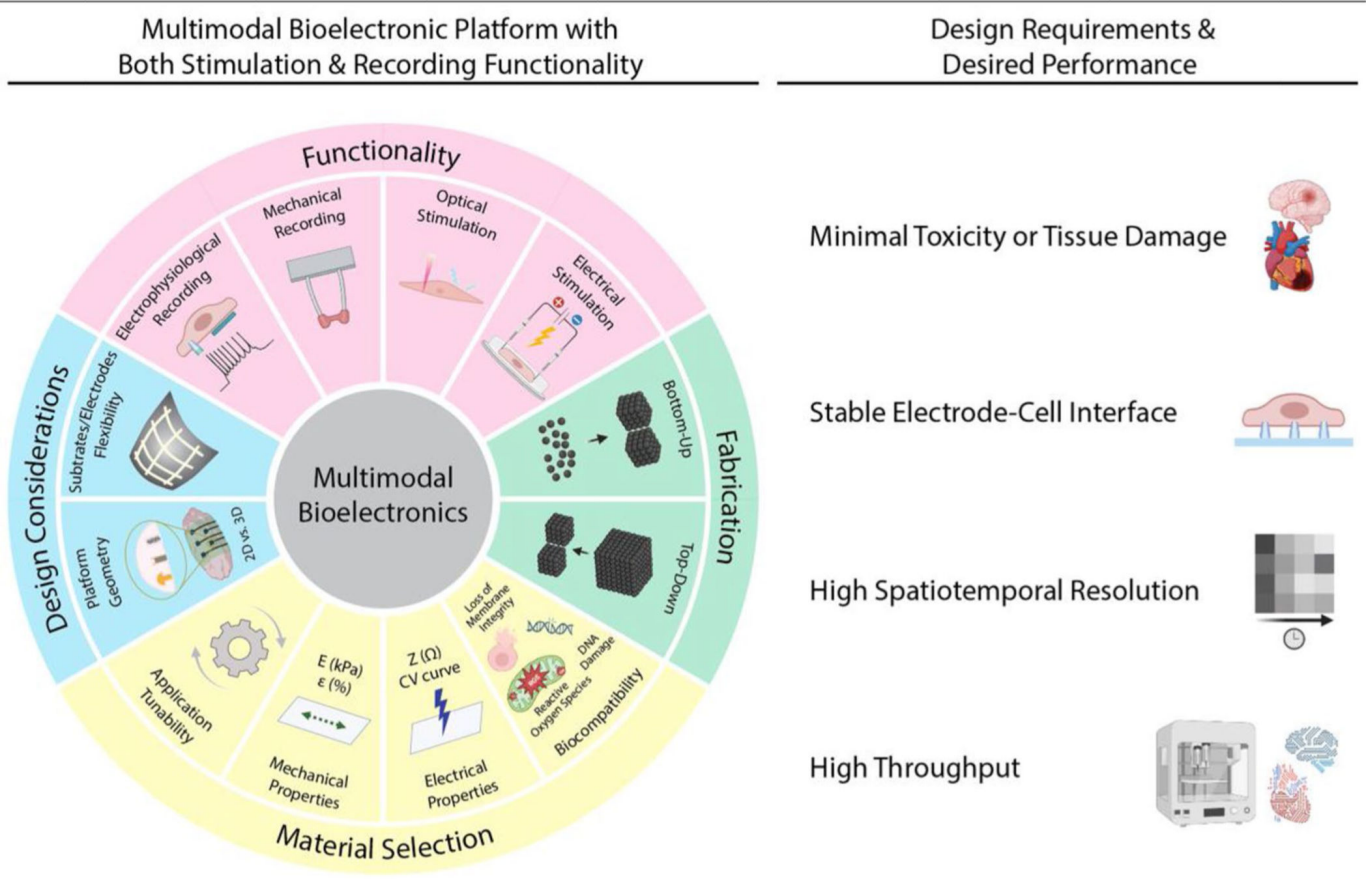
An overview of the functionalities and design, material, and fabrication considerations of multimodal bioelectronic platforms

We further emphasize the design considerations for rigid or flexible multimodal platforms and the possible fabrication techniques. We finally discuss the future directions for multimodal platform development, especially in material development, and the perspective for future platform integration. Through this, a route is highlighted toward achieving widespread high-throughput, high-performance multimodal bioelectronic devices (Fig. 1).

### Electrophysiological and mechanical recordings

Monitoring the electrical activity of cardiac and neural tissues is key to studying changes in ionic fluxes in these cells, namely Na^+^, Ca^2+^, and K^+^, as these ions and respective ion channels are crucial to proper electrical functions and have also been found to be highly impacted in neural or cardiac disorders [7]. In addition to electrophysiology, the electromechanical properties or contractile functions of cardiomyocytes are also important for understanding cardiac function in conjunction with electrical signals due to the significance of excitation–contraction coupling in heart function [7]. Here, we review recent advancements in materials used for these two functionalities.

### Electrophysiological recording

**Fig. 2:**
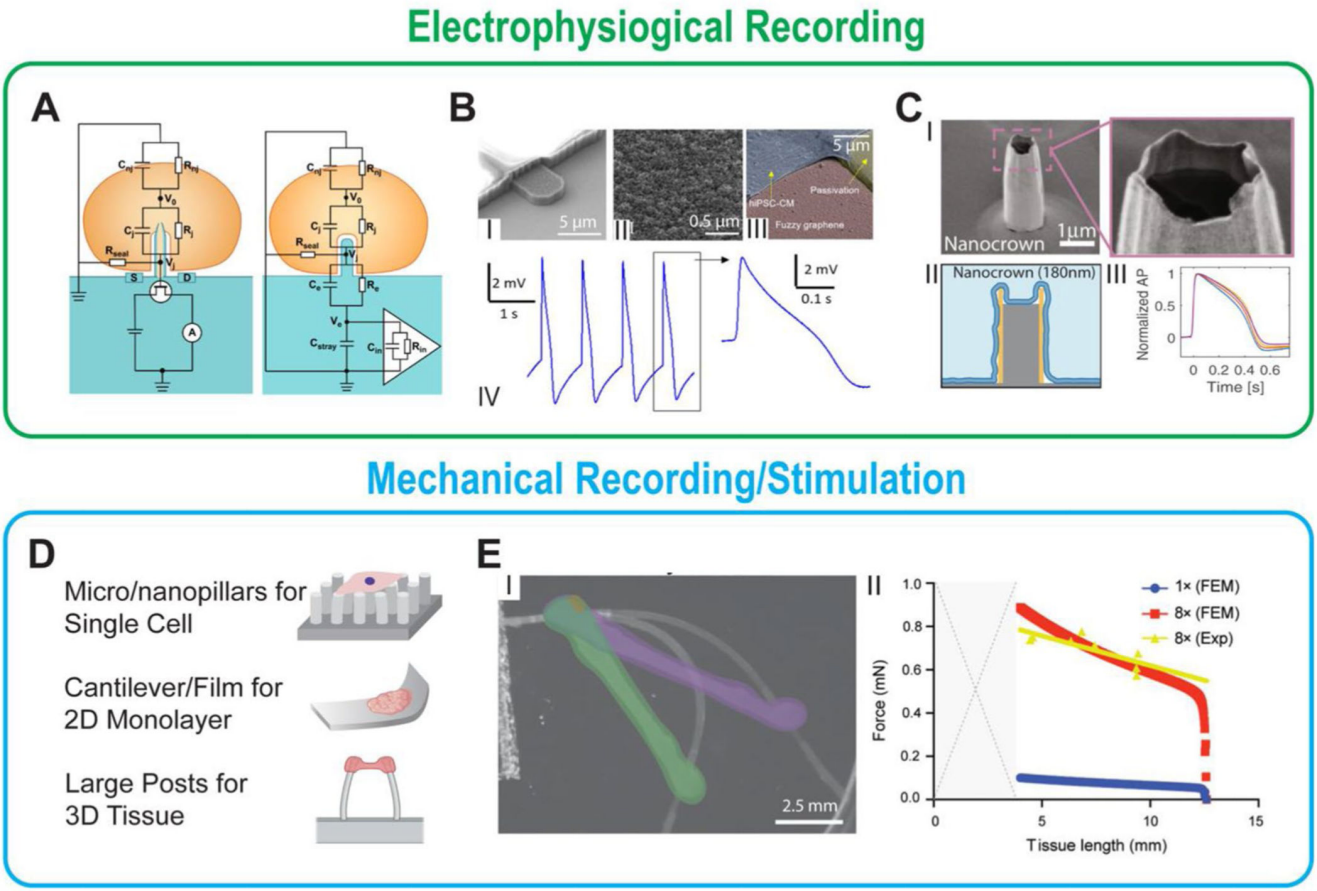
Recent platforms and materials for **A**–**C** electrophysiological and **D**–**E** mechanical recording. **A** Circuit model describing the recording mechanisms using (left) active field-effect transistors (FETs) or (right) passive nanoelectrode arrays (NEAs), applicable for both intracellular and extracellular recording. **B** (I–III) SEM images of 3-dimensional fuzzy graphene (3DFG) electrodes for intracellular recording via optoporation. (IV) Intracellular action potential recording on 3DFG-microelectrode arrays with 50 μm electrodes after optoporation (n = 70 electrodes). **C** Nanocrown NEAs via electroporation with > 99% success rate of intracellular access. (I) SEM image of a nanocrown electrode. (II) Stable interface of nanocrowns with the cell membrane. (III) Aligned intracellular action potentials showing distinct durations and waveforms for cardiomyocytes in the same culture. **D** Schematic overview showing the assessment of contractile functions of in vitro cardiac models in the forms of single cardiomyocytes, 2-dimensional (2D) cell monolayers and 3D cardiac models using nanopillars, cantilevers, and posts, respectively. **E** Engineered heart tissue fabricated around polydimethylsiloxane (PDMS) strips for mechanical loading and measuring tissue contractile force. (I) Macroscopic tissue contractions overlaid with tissue in systole (green) and tissue in diastole (purple). (II) Average force values obtained at each tissue length with either the 1 × or 8 × strips. **A**, **B**, **C** and **E** are adapted with permission from [11–13, 36], respectively

Electrophysiological recordings are used to analyze the changes in the amplitude and duration of ionic transients as well as signal propagation trends across a cell network [7]. The material system chosen can be used to make active devices, such as field-effect transistors (FETs), or passive devices, such as microelectrode arrays (MEAs) (Fig. 2A). Both active and passive devices can be used to record extracellular and intracellular recordings which can be utilized to investigate the spatiotemporal changes to electrical activity of neurons and cardiomyocytes. Extracellular recordings, analyzing extracellular field potentials, offer advantages of non-invasiveness while recording ionic transients detectable across the electrode–cell interface [10]. Intracellular recordings, analyzing action potentials, are able to detect subthreshold potentials with direct access to the cytoplasm of the cell [11]. Recent studies have therefore been aiming to combine the advantages of both, namely utilizing noninvasive extracellular methods to detect intracellular-like signals using electrode materials and fabrication schemes to increase the seal resistance from extracellular recording platforms [12]. The macro/micro-scale device geometry and the nanoscale material topology and effective surface area coupled together allow for electrophysiological recordings with enhanced seal resistance, minimizing signal loss, and therefore, improving signal fidelity [13]

For spontaneous intracellular access, nanotopography of electrodes largely influences the electrode–membrane adhesion and thereby interface stability [13]. Therefore, various 3-dimensional (3D) shapes have been used for intracellular recording, from nanopillars or nanotubes [14] to U-shaped [15], branched [16], or kinked nanowires [17], as well as more complicated shapes such as mushroom [18], volcano [19], and crown [12]. For aided intracellular access mechanisms, such as electroporation [20], optoporation [21], and surface chemical modification [19, 22], both electrode geometries and material selection can improve penetration efficiency, further increasing signal-to-noise (SNR) ratio [23]. For example, electrodes that use 3-dimensional fuzzy graphene (3DFG) benefit from its high surface area and thus require lower laser intensity for optoporation, achieving lower invasiveness (Fig. 2B) [11, 16].The semihollow nanocrown electrodes (Fig. 2C) better stabilized the biointerface than the previous nanopillar design [12], achieving 99% electroporation success rate and prolonged stable access [12].

Across the field of materials used in electrophysiological recordings, materials selection is driven both by the dimensionality of the electrode and by the interface between the electrode and the target cells. 3D electrodes which aim to form interfaces with high seal resistance must be capable of conformal deposition or synthesis on an existing electrode with non-damaging conditions (high temperature, corrosive precursors, and harsh solvents). 2-dimensional (2D) planar electrodes provide more flexibility with materials selection, primarily from their compatibility with traditional thin-film technologies. The base metallic layer in such electrodes can consist of a range of conductive, biocompatible materials, including Au, Pt, graphene, and 3DFG [3, 24]. From this base layer, secondary coatings are applied to reduce electrode impedance, the most common of which include (3,4-ethylenedioxythiophene) polystyrene sulfonate (PEDOT:PSS), platinum black, and TiN [3, 24]. In contrast to these two established schemas for electrophysiological recording, novel material systems such as material inks or liquid metals are being developed through bottom-up synthesis processes aiming for comparable recording quality with greatly suppressed cost and fabrication time [25, 26]. Therefore, the intended fabrication method (bottom-up or top-down) and the intended cell–electrode interface and resultant type of recording (intracellular or extracellular) should dictate the material system choice in electrophysiological devices.

In conjunction with electrophysiological recordings, simultaneous optical mapping using voltage-sensitive dyes (VSDs) or voltage-sensitive fluorescent proteins (VSFPs) offer a non-invasive, high-resolution, and scalable technique for investigating ionic fluxes present during cellular electrical activities in 2D cell cultures [27]. VSDs change their molecular structure and fluorescence response with the surrounding electric field to detect millisecond action potential changes [28]. Furthermore, optical recording provides the opportunity to individually map ion specific transients, the most widely investigated being Ca^2+^. For instance, the monitoring of Ca^2+^ in cardiomyocytes has been achieved using Ca^2+^ fluorescence indicators allowing for understanding the ion transition dynamic during the excitation–contraction coupling [10]. The isolation of a singular ion transient, however, does not yield information on subthreshold values indicative of cellular processes which drive the voltage above threshold values, driving the need for voltage sensitive and not ion sensitive dyes which are more sensitive and of reduced toxicity [27, 29]. Additionally, optical imaging techniques require highly specialized equipment to capture images at frame rates comparable to the sampling frequency of electrophysiology recording systems, placing a barrier on the widespread use of highly developed techniques using VSDs or VSFPs.

VSDs are also currently limited in 3D volume-based imaging, as the image is typically focused on a single plane while detecting changes in fluorescence intensity, though the 3D imaging field has made tremendous progress in recent years [30]. Therefore, while VSDs and VSFPs can enhance our understanding of disease progression or functional changes in excitable cells, electrophysiological measurements offer an advantage when looking at multiple ion transients at once and while investigating 3D tissues, while maintaining high sensitivity and low toxicity.

### Mechanical recording

Evaluation of mechanical motion or contractile function of cardiomyocytes is critical to study heart function as contractile dysfunction is key to the heart failure pathobiology [31]. Numerous methods have been employed with variable device geometry and detection methods (Fig. 2D). The most used principles include analyzing high frame-rate videos capturing the deformation of flexible pillars or cantilevers/thin films and converting motion to force measurements based on the elastic modulus and dimensions of the material and include using interdigitated electrodes for impedance-based mechanical recordings [32–34]. An expanding method of mechanical recordings utilizes materials which convert applied stresses to electrical or optical information. Integrating these materials into pillars or cantilevers has demonstrated improved measurement accuracy, real-time monitoring, and visualized contraction [32–34].

Force/strain measurements have been utilized in work of Zheng et al., which used piezophototronic InGaN/GaN nanopillar arrays to visualize the cardiomyocyte contraction through changes in photoluminescence intensity or displacement in the tips of the nanowires, exhibiting 800 nm resolution and subcellular mapping of multidirectional forces [32]. Novel material strain gauge sensors have been engineered using biohybrid 3D printing of a carbon black polydimethylsiloxane (PDMS) cantilever together with engineered heart tissue [34]. Additionally, photonic-based devices such as anisotropic gelatin methacryloyl (GelMA) hydrogels with super-aligned carbon nanotube sheets and non-close-packed colloidal arrays of silica nanoparticles, allow for direct visualization of cell mechanical motion based on the phenomena of structural color materials [33].

Mechanical recording devices vary in composition based on the type of tissue under investigation and mechanical recording mechanism (piezophototronic or displacement monitoring) [32, 34]. Interdigitated electrodes or cantilever-type devices can be made from Au, PEDOT:PSS, platinum black, or phototronic materials, whereas more complex devices like nanopillars require materials which can be fabricated in 3D [32–34]. The mechanical properties and compatibility with the target biological systems are key factors to consider when selecting materials for the substrate. Therefore, the overall device design is the fundamental consideration for selecting what type of material is used for the mechanical recording.

A side from mechanical recording, mechanical stimulation is also an important element in closed-loop multimodal platforms because it has the capability to model the developmental paradigm and improve in vitro tissue function [35, 36]. Simple platforms such as a PDMS strip, incorporated by Bliley et al., can be used to measure tissue contractile forces, provide mechanical loads, and model hemodynamic loading on engineered heart tissues to recapitulate disease phenotypes (Fig. 2E) [36].

### Electrical and optical stimulation

Electrical stimulation has been clinically used for disease intervention, such as deep brain stimulator for Parkinson’s disease [37] and implanted pacemaker for arrhythmia [38]. Modulating the electrophysiology allows researchers to investigate the working principles and pathways of neural and cardiac structures from single-cell level to organ and systems [39]. The modulation can be accomplished through electrical stimulation by injecting charge using bioelectronic devices [3, 40], or optical stimulation by affecting the ion channels (e.g., light-sensitive proteins or voltage-gated proteins) or changing cell membrane properties with incident light [41–43]. Here, we review the recent progress of materials utilized for both electrical and optical stimulations.

### Electrical stimulation

**Fig. 3:**
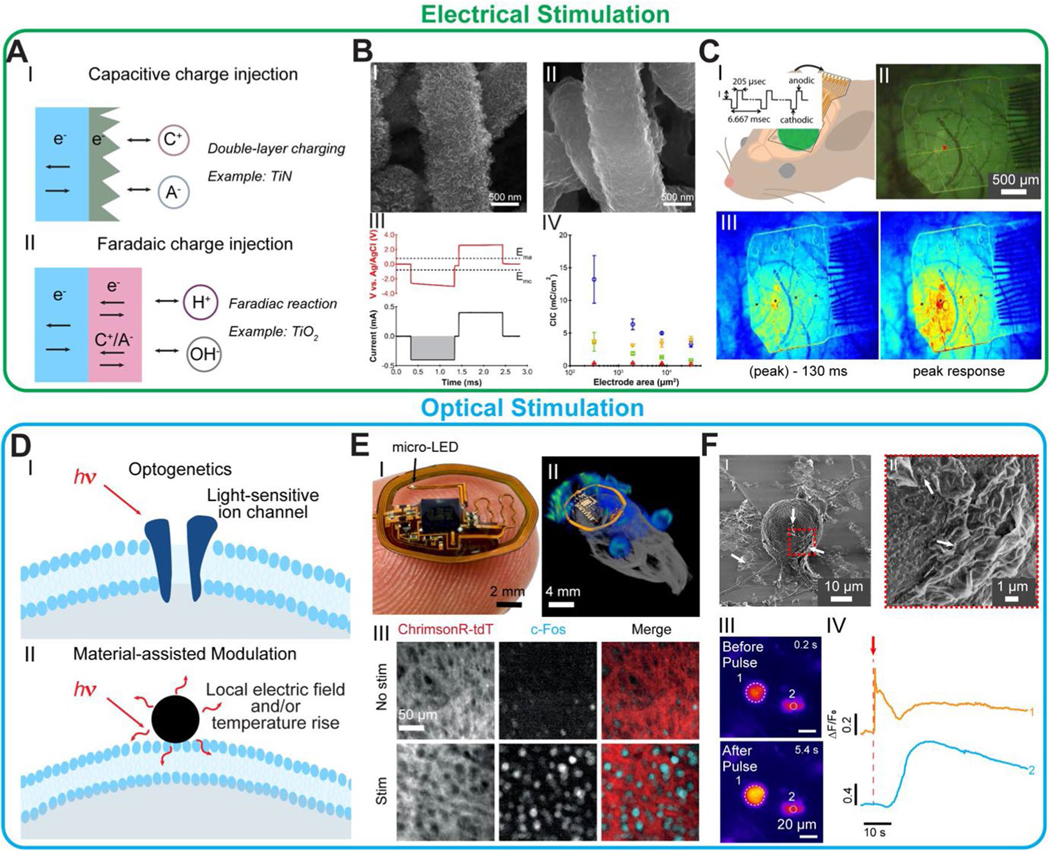
Recent platforms and materials for **A**–**C** electrical and **D**–**F** optical stimulation. **A** Schematic of charge injection mechanisms. **B** Representative scanning electron microscopy (SEM) images of (I) nanowire-templated 3-dimensional fuzzy graphene (NT-3DFG) and (II) NT-3DFG + poly(3,4-ethylenedioxythiophene) polystyrene sulfonate (PEDOT:PSS) (III) Representative voltage transient (red) of a 200 μm NT-3DFG + PEDOT:PSS measured as a response to applied biphasic current pulse (black). The shaded region denotes the charge injected into the electrode/electrolyte interface. (IV) Charge injection capacity (CIC) characterization of Pt (red triangles), PEDOT:PSS (yellow triangles), NT-3DFG (green boxes), and NT-3DFG + PEDOT:PSS (blue circles) microelectrodes as a function of the geometric area of the electrodes. Results are presented as mean ± SD (n = 5 electrodes per material and geometry with 10 pulses per current amplitude). **C** (I) Schematic of microelectrocorticography (ECoG) implantation over sensorimotor cortex and electrical stimulation in GCaMP6f mice. (II) Visualization of the fluorescent neural response after stimulation with a single graphene electrode site (marked with a red triangle). (III) Visualization of the intensity of neural response to 500 μA electrical stimulation at times—130 ms before peak response and peak response with the same graphene electrode array as in (II). **D** Schematic of genetic and non-genetic optical modulations. **E** (I) Photographic image of device balanced on a finger. (II) Combined magnetic resonance imaging (MRI) and computed tomography (CT) 3D rendering of a device implanted in a mouse. (III) Example images of c-Fos staining in M2 of mice expressing ChrimsonR-tdT with or without transcranial optogenetic stimulation. **F** (I) Representative SEM image of a dorsal root ganglion (DRG) neuron incubated with 100 μg/mL Ti_3_C_2_T_*x*_ flakes. White arrows denote Ti_3_C_2_T_*x*_ flakes interfaced with the neuron and dispersed on the substrate. (II) Expanded view of the marked red dashed box in I. (III) Time series fluorescence images of a representative DRG neuron labeled with a Ca2 + indicator (CalBryte 520 AM). A 635 nm laser pulse of 18 mW power and 1 ms pulse duration was applied at t = 5.0 s. (IV) Normalized Ca^2+^ fluorescence intensity as a function of time for the ROIs marked in III. The red arrow denotes the starting point of the applied laser pulse (*t* = 5.0 s). **B**, **C**, **E**, **F** are adapted with permission from Refs. [24, 46, 48, 54], respectively

Electrical stimulation modulates electroactive cells by depolarizing the cell membrane interfaced with the electrodes [3, 40].The current or voltage waveform is applied to the stimulation electrodes and generates an ionic flow near the cell membrane to induce cell depolarization [3, 40]. The charge injection mechanism at the electrode–cell interface includes capacitive and faradaic injection depending on the material type [3]. The capacitive charge injection is governed by the double-layer ion–electron charge separation, and the faradaic charge injection involves the chemical reactions between cations or anions with the electrode materials (Fig. 3A) [3, 40]. The mechanism of charge injection has implications for the long-term stability of the electrodes and the potential toxicity to the environment due to the formation of by products [3, 40].

Ideally, a material used for electrical stimulation is expected to have a high charge injection capacity (CIC), high charge storage capacity (CSC), long-time stability, and biocompatibility to ensure a safe charge injection without electrode degradation or cell damage [3, 40]. Noble metals and metal oxides, such as Pt [24] and I rO_x_ [44], have been clinically approved for electrical stimulation [24, 44]. Advanced materials and structures, such as nanocarbons [24] and conductive polymers [45], have shown enhanced charge injection capacity, biocompatibility, and mechanical compliance to improve the performance and stability of the stimulation. Garg et al. demonstrated that high surface area nanowire-templated 3DFG (NT-3DFG) showed a CIC of 3.66 ± 1.42 mC/cm^2^, which is 35-fold higher compared with conventional Pt and Au metal electrodes (Fig. 3B) [24]. To investigate the neural or cardiac behavior during electrical stimulation, electrodes capable of optical imaging or magnetic resonance imaging have been developed [46]. Park et al. reported a patterned highly transparent and artifactfree graphene electrode array with a CIC of 57.13 μC/cm^2^ and showed adaptability with real-time fluorescence imaging (500 μA current injection) (Fig. 3C) [46]. These material engineering advancements have enhanced electrode properties, including optical and electrical characteristics, enabling reduced footprint, improved stability, biosafety, and compatibility with various modalities, e.g., fluorescence imaging.

### Optical stimulation

Remote optical-based cell stimulation is an emerging approach that offers high spatiotemporal resolution, reduced invasiveness, and the advantage of avoiding electrical artifacts for simultaneously recording and stimulation [41–43]. Optogenetics is a breakthrough technology that utilizes genetically transfected cells expressing light-responsive proteins in the membrane (Fig. 3E, I) [43]. This technique has been widely used in investigating pathways related to neurological diseases [39] and constructing biohybrid robots [47]. The development of light delivery approaches, including high-density μ-LED arrays [48] and flexible photonic waveguides [49], has facilitated targeted light delivery to specific tissues. The integration of optical electronics and optogenetics has demonstrated successful stimulation capability in free-moving animals [48]. For instance, Ausra et al. developed battery-free and wireless communicable μ-LED implants to achieve subdermal optogenetic stimulation of the secondary motor cortex in freely moving mice, indicated by the neural excitation fluorescence indicator c-Fos (Fig. 3F) [48].

Light-responsive materials provide non-genetic opportunities for optical stimulation of cells and tissue. During light illumination, these materials will generate local temperature rises and the electric field that will change the interfaced cell membrane properties, resulting in cell excitation or inhibition (Fig. 3E, II) [41, 42]. Various photo transducers, including Au- [50], Si- [51], C- [52], and polymer-based [53] materials, have been utilized in optical stimulation. These transducers exhibit photothermal, photoelectric, or photomechanical responses [50–54]. To ensure the minimal negative impact on the stimulated cells and tissues, it is crucial for the photo transducers to possess high near-infrared absorption, and high light conversion efficiency to enable the low incident energy for cell stimulation. Recently, Wang et al. demonstrated that Ti_3_C_2_T_*x*_ (MXene) is an outstanding candidate for achieving neural stimulation with less than μJ incident energy without causing irreversible damage to the interfaced cells (Fig. 3G) [54]. Engineering the size, composition, and surface chemistry of the transducers presents as possible strategies to further enhance the light absorption and conversion [42, 50, 53]. Additionally, incorporating binding agents and biocompatible coating holds promise to enhance the stability and biocompatibility of material–cell interface [42, 50].

### Comparison of electrical vs. optical stimulation

The selection between electrical and optical stimulation methods highly depends on the desired spatiotemporal resolution, the specific cell or tissue type of interest, and the tolerance for invasiveness. Electrical stimulation platforms demonstrated impactful contributions to therapeutics interventions and understanding of neuroscience with a versatility of engineering flexibility, such as using shank-type or syringe-injectable electrodes tailored for brain or cardiac regions of interest [55]. Such platforms, while straightforward to develop and apply, face limitations in their necessity to be tethered transdermally or requirements for embedded power supplies [55–57]. On the other hand, optical stimulation bypasses the tethered restriction by achieving cellular stimulation with remote light sources. With its superior temporal and spatial resolution when applied in conjunction with nanotechnology, precise stimulation of individual cells is possible with well-timed laser pulses [42]. The translation of optical stimulation with materials in vivo demonstrates potential for remote, non-invasive control of cellular activity without the tethering restriction; however, the appropriate optical hardware for effective delivery of light in deep tissue and targeted region still requires further investigation [42]. Hence, both methods offer distinct advantages and drawbacks, prompting researchers to customize their approaches based on their specific application and desired outcomes.

### Materials and design considerations in future multimodality

**Fig. 4:**
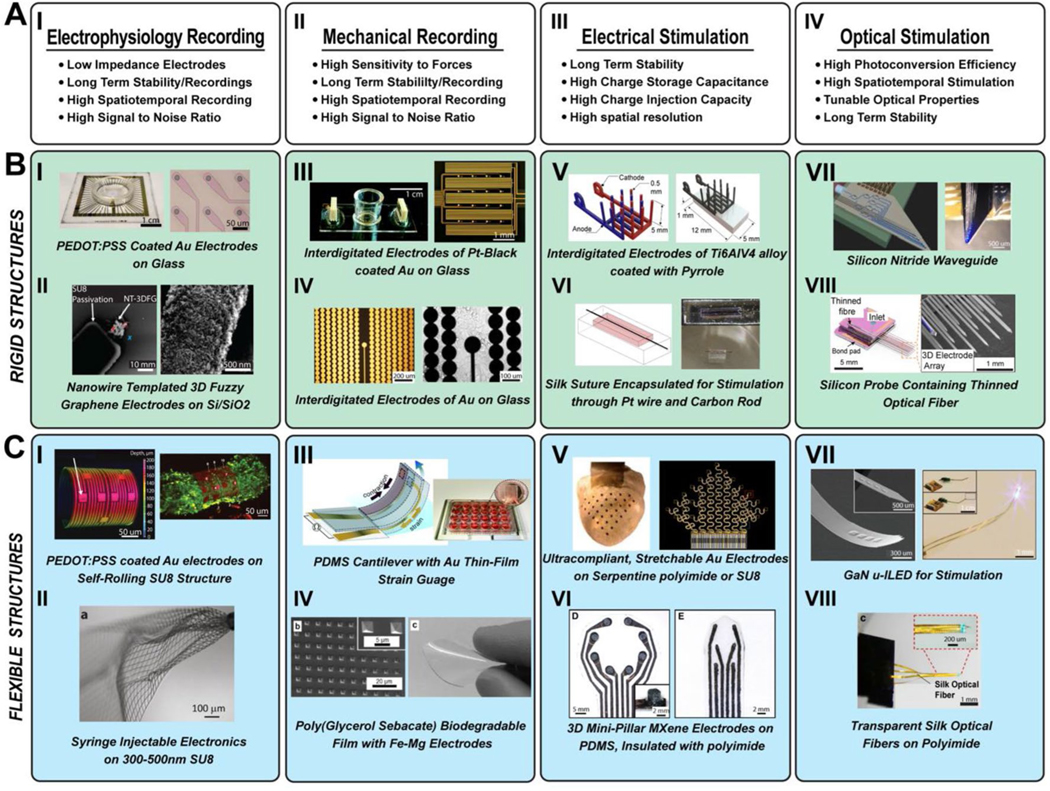
Design considerations in geometry and substrate across varying functionalities. **A** Design requirements for (I) electrophysiological recording platforms, (II) mechanical recording platforms, (III) electrical stimulation, and (IV) optical stimulation. **B** Current platforms and materials on rigid substrates for each bioelectronic modality. (I) Optical image of transparent microelectrode array (MEA) made of poly(3,4-ethylenedioxythiophene) polystyrene sulfonate (PEDOT:PSS) coated Au electrodes. (II) Scanning electron microscopy (SEM) image of nanowire-templated three-dimensional fuzzy graphene (NT-3DFG) ultra-microelectrode and expanded view of the red dashed box. (III) Schematic of chip and optical image of microelectrode sensor arrays. (IV) Microscope image of the integrated device with interdigitated electrodes, reference electrodes, microelectrodes, and an image of cells cultured on the device. (V) Schematic representation of 3D electrode units in interdigitated design and render of coated electrodes as assembled in 3D. (VI) Schematic of biowire in cell suspension and biowire in human embryonic stem cell-derived cardiomyocytes (hESC-CM) suspension. (VII) Schematic showing fiber and electrical packaging on printed circuit board of neural probe and microscope image of a device with emitters and optical output. (VIII): Photograph of 3D multifunctional MEA and SEM image of 3D electrode array and optical fiber. **C** Current platforms and materials on flexible substrates for each bioelectronic modality. (I) 3D confocal image of 3D self-rolling biosensor array (3D-SR-BA) with microelectrodes and confocal image of 3D cardiac spheroid with calcium indicator dye encapsulated by 3D-SR-BA. (II) Image showing the mesh electronics was injected into 1 × phosphatebuffered saline (PBS) solution by a glass needle with inner diameter (ID) of 95 μm. (III) Sketch of device cantilever and example 24-well device for parallel experiments. (IV) SEM images of microstructured poly(glycerol sebacate) (PGS) films with 2D array of square pyramids in the PGS film and an image of a PGS transparent biodegradable elastomer film. (V) Graphical depiction of 3D multifunctional integumentary membranes (3D-MIM) on heart model and serpentine structure of 3D-MIM. (VI): Images of MXtrode array used for electrocorticography (ECoG) and electrical stimulation. (VII): SEM of an injectable array of micro-inorganic light-emitting diodes (μ-ILEDs), total thickness, 8.5 um, and integrated system wirelessly powered. (VIII): Microscope image of ultrathin flexible electrode array and photograph of Silk-Optrode probe connected to laser light source. **B** and **C** are adapted from Refs. [10, 23, 55, 59–65, 72, 80–84], respectively

Stimulation and sensing/recording nodes serve as fundamental components for multimodal platforms. To effectively design such platforms, it is essential to establish guidelines and criteria based on the intended application of different materials. The overall functionality and application of the platform rely on the strategic integration of these materials and modalities, considering specific requirements such as substrate type, size, and geometry, as well as electrode characteristics like material type, CIC, CSC, and spatial resolution (Fig. 4).

#### Rigid surfaces

Historically, bioelectronics have been fabricated on rigid surfaces using well-developed micro- and nanofabrication techniques, e.g., physical and/or chemical processes to form electrodes and their passivation layers [58]. Photolithography done on rigid surfaces allows patterning of (soft) materials in a well-defined manner (e.g., conductive polymers) (Fig. 4B, I–IV, VII, VIII) [23, 59–63]. Materials such as glass or Si/SiO_2_ (Fig. 4B) are common surfaces for bioelectrical devices compatible with traditional lithography methods. Bioelectronic devices fabricated on rigid surfaces enabled extensive investigations of cultured 2D cell monolayers [23, 59–61]. Transparent electrodes for 2D cell cultures have allowed researchers to investigate electrical signals during optical imaging, such as Ca^2+^ imaging (Fig. 4B, I) [59]. Mechanical recording utilizing interdigitated, impedancebased sensing on rigid surfaces, e.g., glass, has been extensively demonstrated (Fig. 4B, III–IV) [23, 61]. Rigid devices have been used for electrical stimulation for neurite growth (Fig. 4B, V) [64] or cardiac tissue maturation (Fig. 4B, VI). [65] 3D rigid structures have been deployed in chronic bioelectronic devices [66]. Shank-type platforms (e.g., Michigan probe) are commonly used for brain neural electrical activity recording and electrical and optical stimulation (Fig. 4B, VII––VIII) [62, 63]. Rigid surfaces enable bioelectronics materials development by allowing elevated temperatures needed in synthesis processes such as chemical vapor deposition. Recently, NT-3DFG, a unique hybrid-nanomaterials used in bioelectrical sensing and stimulation was demonstrated via an elevated temperature chemical vapor deposition (CVD) synthesis process (Fig. 4B, II) [60].

#### Flexible surfaces

Flexible platforms for bioelectronic devices are motivated by the mechanical mismatch between the interfaced rigid devices and the soft tissue or cell culture [67]. This mechanical mismatch can cause inflammation and/or a foreign body response in vivo, which can lead to fibrotic tissue formation around the device resulting in reduced device recording and/ or stimulation yield [67]. Flexible bioelectronics are defined as devices which under mechanical stresses or strain, maintain their material properties, mainly electrical conductivity, and impedance [67].The development of flexible bioelectronic devices has grown significantly in the past decade, aiming to gain information about a biological system in its native conformation or to improve the benchtop to clinical use of a device [56]. Initial development on flexible devices utilized similar fabrication techniques discussed above.

#### Folding/curling/compacting

Flexible structures have been integrated with cardiac and neural 3D engineered tissue (e.g., spheroid, organoid) in vitro. Self-rolling (folding) structures have been produced such that 3D tissues are encapsulated within a device (Fig. 4C, I, III) [10, 68]. The use of different 3D shapes and platforms has allowed for interfacing with engineered tissues for more physiologically relevant models and has expanded the tools available for recording and stimulation. Engineering to allow for this type of shape change has been seen using pre-stressed metallic films for self-rolling devices, prestressed PDMS which when resolved renders a 3D platform or through using varying thicknesses of a SU8 insulation layer and specific release procedures [10, 68, 69]. Tuning the properties of the shape change against other structural properties such as porosity is necessary based on the end application.

#### Stretchable

Stretchable devices are necessary in applications where movement in more than one dimension could cause fracture of non-stretchable metals or insulation layers. Kirigami inspired geometries are used to extend 2D rigid, precursor geometries into a stretchable device [70]. Kirigami-based approaches vary widely in the types of geometries from uniaxial and biaxial serpentine layouts to concentric arc designs for extension into 3D. This technique allows for application-based tunability to create a stretchable platform with specific properties to allow for anticipated stresses (Fig. 4C, II, IV, V) [70–72]. Numerous research teams have employed serpentine structures or pre-stretched devices such that the devices can withstand multiple cycles of movement in all directions without device failure [73]. These devices have allowed for a stretchability of over 100% or higher based on the pre-stressed conditions or the extent to which the serpentine structure of the device is laid out and can be tuned based on the anticipated mechanical stresses in use.

Stretchable platforms can also be engineered using flexible or stretchable materials without compromising the conductivity of the material [74, 75]. Stretchable hydrogels used with liquid metal-based electrodes have allowed for soft elastomers to be interfaced with eutectic gallium indium alloy for wearable devices with up to 820% stretch with minimal increase in resistance [76]. Other methods include using tough hydrogels with silanized titanium wire to engineer stretchable, biocompatible electrodes [77].

#### Ultra-compliant

The development of ultra-compliant platforms for bioelectronic devices is motivated by a closer tissue to device spacing for improved recording and stimulation capabilities [78]. Compliant electronics can conform to the curvature of a 3D interface, whether an organoid system in vitro, or a wearable, skin-based electronic platform, allowing for a minimal interference with normal cell or tissue function [73, 79].

Ultrathin materials, such as SU8 or polyimide, have been used to create ultra-compliant devices which conform to the heart or brain tissue for the smallest electrode–cell interface, improving the recording capabilities or the stimulation efficiency across the interface. Ultrathin materials can therefore be injected into the body without requiring highly invasive procedures (Fig. 4C II) [55]. Devices which are ultra-complaint can be characterized based on Young’s modulus between 1 and 100 kPa and are frequently developed in the form of microelectrode arrays [67], greatly reducing the size and eliminating the rigidity of other platforms, not only eliminating challenges associated with the electrode–cell interface but also minimizing the potential for long-term inflammatory responses (Fig. 4C, V–VIII) [15, 80–82].

### Material selection and fabrication process

As the demands for bioelectronics devices grow in multimodality and materials systems become more complex, manufacturability, both at the laboratory and at the commercial scale, becomes an increasingly more pertinent consideration. This is compounded by manufacturing methods for many high-performing materials, many of which are intrinsically incompatible, especially limiting the potential for multimodal devices. These issues bring the methodology in device manufacturing into focus, both in their technologies, versatility, advantages, and limitations.

### Top-down fabrication

**Fig. 5:**
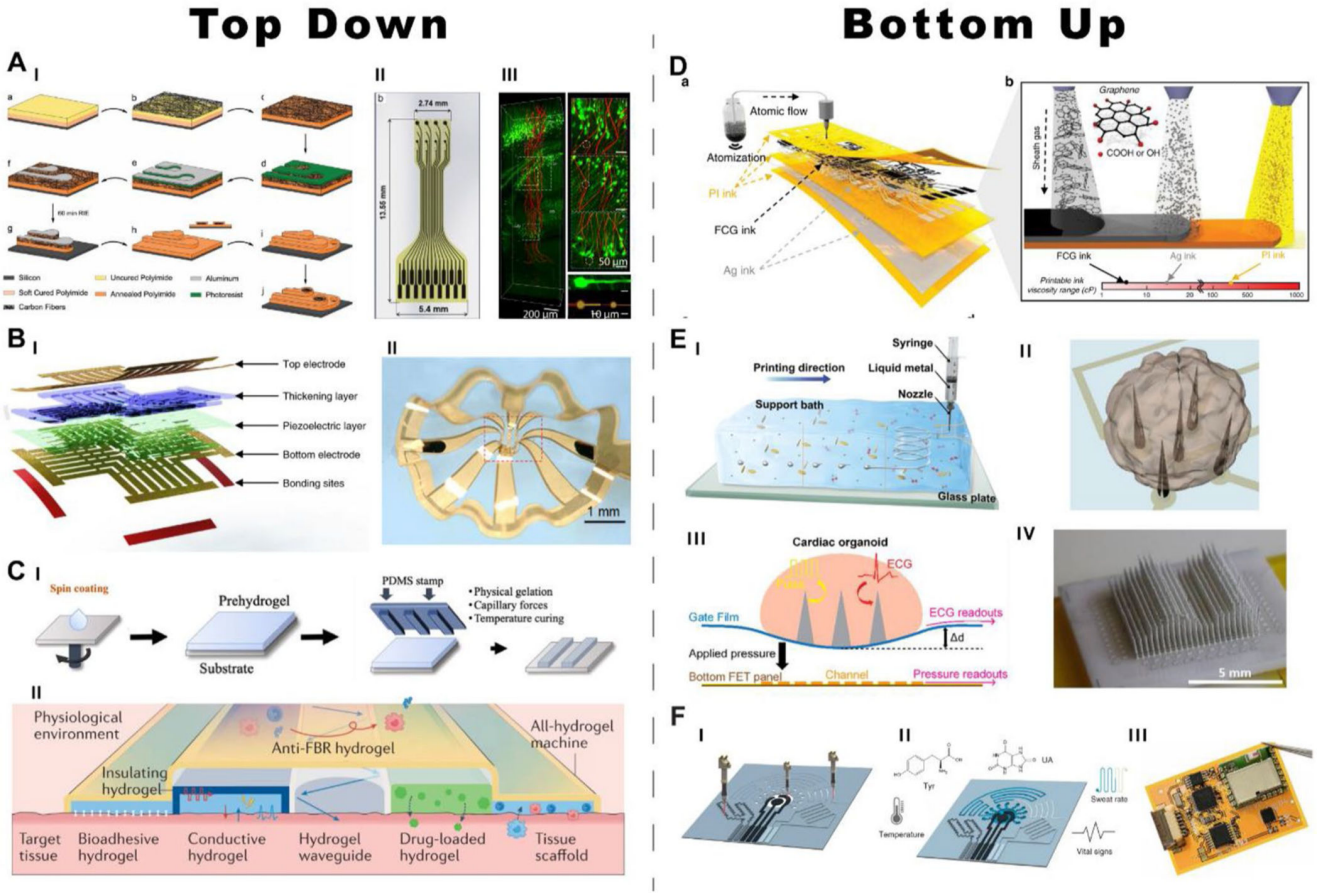
Manufacturing techniques for multimodal bioelectronic devices. **A** Planar device configuration with fabrication schematic (I) and final device geometry/scale (II), reproduced with permission from Ref. [97] with an additional application (III) for neuron-scale, injectable electrodes systems, scanning electron microscopy (SEM) and fluorescence microscopy images adapted from Ref. [15]. **B** Self-buckling mesostructures from planar structures with representative device stack (I) and example resulting mesostructure (II) adapted from Refs. [73] and [85]. **C** Hydrogel-based planar devices with a soft lithography-based patterning schematic (I) and an example of an all-hydrogel multimodal device adapted with permission from Ref. [98, 99]. **D** A sequential deposited planar ink-based bioelectronic device with multiple functional inks, adapted from Ref. [25]. **E** An extrusion-based device manufacturing applying liquid metal with a gel support-based extrusion scheme (I) and free-standing extruded liquid metal electrodes incorporated into a multimodal platform (II–IV) adapted with permission from Ref. [26] (I) [93], (II–III) [87], and [26] (IV). **F** Schematic for fabrication of laser-induced graphene devices with multifunctional coatings and final device adapted from Ref. [95]. **A**, **B**, **C**, **D**, **E**, **F** are adapted from Refs. [15, 25, 26, 73, 85, 87, 93, 95, 98, 99], respectively

The primary foci of bioelectronic device manufacturing fall into two paradigms – top-down and bottom-up fabrication. In top-down, hierarchically deposited materials are subtractively removed to form patterned device stacks. In the most traditional form, devices are manufactured through photolithography on rigid or flexible substrates for planar devices (Fig. 5A, I–II). Owing to its versatility across a diverse range of materials, this method has seen the greatest usage in bioelectronics to date, with applications ranging from flexible platforms for developmental characterization of cortical spheroids [73] to closed-loop neuromodulation devices with built-in wireless backend [57]. Since photolithography is primarily a 2D patterning technology, many devices fabricated with this method remain planar and capable only of interfacing with a 2D tissue space. Thin-film devices designed for conformality or injectability (Fig. 5A, II–III) are often applied to suppress this limitation; however, it can be further surpassed through specific geometries and integration of stress by the inclusion of thickening layers in device stacks that allow for the formation of buckling and 3D device geometries (Fig. 5B, I–II). This technique has been simultaneously applied with photolithographic methods for multisensor integration widely in the monitoring of organoid development with the 3D spatial resolution with the integration of electrical stimulation and recording alongside mechanical, temperature, and oxygen monitoring [73, 85].

Interface integrity between materials with different elastic moduli remains a challenge, motivating the use of materials with comparable elastic moduli to avoid damage to soft tissue in application [78]. Ultra-compliant materials such as hydrogels are popular candidates for eliminating the moduli gap between bioelectronics and tissue but are among the limiting options for multimaterial devices given their vacuum/ temperature sensitivity and processing conditions. Despite this, top-down techniques have been adapted where devices are fully encapsulated or cast with patterned hydrogels (Fig. 5C, I) [86] for the integration of hydrogels on existing devices to preserve the multimodal versatility of topdown processing while granting the enhanced tissue-device interface from hydrogels. In addition, hydrogels themselves display a great deal of application tunability, with some compositions displaying enhanced ionic conductivity, low optical absorption, drug-bearing capabilities, and insulating ability allowing for the direct sequential fabrication of multimodal all-hydrogel devices (Fig. 5C, II). The combination of these top-down methods allows for the manufacturing of materially complex and multimodal bioelectronic devices but is not a universal solution to manufacturing. The topdown approach requires high-cost equipment for thin-film processing and, despite the potential for self-buckling structures, is limited to planar device structures. Here exploration of alternate fabrication methods is necessary.

### Bottom-up fabrication

Bottom-up fabrication poses a new and largely unexplored area for bioelectronics which promises opportunities for 3D non-planar structures on a scale unattainable through other means with high throughput, and retention of material versatility. The direct printing of inks is one of the strongest routes for highly manufacturable bioelectronic devices. Inexpensive methods of printing have been demonstrated by reconfiguring extrusion-based printers to print custom materials [87] while commercially available inkjet printers have also been used for bottom-up fabrication of devices [88]. For this application, conductive and active inks are directly deposited through the screen, aerosol jet, inkjet, and transfer printing [89]. Through the integration of multiple functional inks, including conductive, nanoparticulate suspensions, enzyme-based, and polymerizing, entire multimodal bioelectronic devices can be manufactured in one roll-to-roll process (Fig. 5D) [89].

Extrusion-based bottom-up fabrication applies viscous or setting precursor inks to fabricate free-standing or matrix-supported 3D structures (Fig. 5E, I, IV). These structures for bioelectronic devices can manifest unique, non-planar geometries to interface with tissue across a 3D space. Precursors ranging from stabilized quantum-dot suspensions to liquid metals to hydrogels [26, 90, 91] can be applied simultaneously to fabricate multifunctional devices either from the ground up or on backend-integrated substrates. This technique has found applications across the full range of bioelectronics, including the fabrication of μ-LED arrays, vertically standing liquid metal electrodes, strain gauges, and biosensors [87, 91–93]. While the combination of structures possible with extrusion-based manufacturing has been demonstrated for the interrogation and modulation of cardiomyocytes, its full potential is still undergoing exploration and represents one of the major frontiers in multimodal bioelectronics.

While extrusion-based and direct printed techniques show great promise in bioelectronic manufacturing, both require that materials are largely synthesized prior. In-situ conversion of materials represents a lower cost and direct method for high-performing bioelectronic devices, the most prominent technique of which is laser-induced graphene. First demonstrated on polymeric substrates in 2014 [14], laserinduced graphene is produced by irradiating a polymer film with a high-power laser, allowing for the direct writing of patterned flexible electrodes. While their first manifestation was for electrophysiology/stimulation, it has since evolved through post-treatment and in-situ nanoparticle synthesis for uric acid, pH, strain, monoamine, temperature, glucose, and more [71, 94–96]. The integration of these mechanisms represents a route to solving one of the most long-standing limitations in multimodal bioelectronics – the restrictively high amounts of time and resources necessary to prototype devices.

Both top-down and bottom-up fabrication techniques present not only options for developing multimodal bioelectronics across 2D and 3D but also as complementary processes for developing powerful, materially complex devices. Exploration of new and promising bottom-up fabrication and its co-application to top-down represents one of the avenues of greatest potential in bioelectronics, particularly as the necessity for conformal, tissue-like, and lifetime-engineered interfaces becomes more prominent.

## Conclusion and outlook

Application-driven multimodal bioelectronic devices are an emerging and powerful approach for disease treatments, wellness monitoring, and basic science research [61, 100]. Here, we briefly summarized the individual components of the platform including recording and stimulation and highlighted the consideration of material selection for improved performance. We reviewed the geometries and designs of the integral platforms from 2D to 3D, and the recent top-down and bottom-up synthesized fabrication procedures.

The materials’ advancements discussed in this review highlight the immense potential of nanomaterials for further research and development in miniaturized sensors, wearable devices, and flexible electronics [8]. Nanomaterials exhibit exceptional properties distinct from their bulk counterparts, displaying remarkable optical, magnetic, catalytic, mechanical, and electrical characteristics. Utilization of these unique properties enables device engineering and communication on the cellular and subcellular size scale for unprecedented control, fidelity, and span in bioelectronics. As the manufacturing techniques for these nanomaterials continue to develop in resolution and scale, including the rapid refinement of ink-based and multimaterial 3D printing [34, 94], an opportunity opens for high-throughput development of integrated bioelectronics in 2D and 3D spaces [14–19]. The diverse applications of these nanomaterials underscore their versatility and transformative potential to be used in future multimodal bioelectronics.

Though the recent advancements in material engineering, platform design, and manufacturing have shown significant progress, the following improvements could be carefully considered for future directions.

1. Minimal toxicity and mechanical mismatch to diminish tissue damage. Beyond conventional Si- and metal-based bioelectronics, recently developed hydrogel and conductive polymers have paved new opportunities for biocompatible interfaces [67]. Engineering morphology, for instance, minimizing the footprint of the electrodes, has also exhibited its promising ability in reducing scar formation after implantation [60].
2. Stable and long-lifetime interface. The degradation of the electrode materials will reduce the performance of the electrode and potentially generate toxic species [3]. Enhancements in material geometry and chemical stability, as well as the improvements in platforms’ stretchability and compliance, enable to establish the interface with prolonged stability [40, 42, 67].
3. High-throughput fabrication for the integral platforms. The investigation of generalized material type and procedure is necessary to achieve high-quality fabrication of multimodality platforms with large-scale production [94].

The success of the in-lab multimodality prototypes has pioneeringly broadened the understanding of tissue and animal models. Overcoming the above-mentioned engineering and material challenges will benefit the platforms for widely clinically and commercial applications.

## Data Availability

Not applicable.
